# Trends in diagnostic nuclear medicine in Sweden (2008–2023): utilisation, radiation dose, and methodological insights

**DOI:** 10.1186/s40658-025-00747-2

**Published:** 2025-04-02

**Authors:** Anja Almén

**Affiliations:** 1 Medical Radiation Physics, Department of Translational Medicine, Lund University, Carl-Bertil, Laurells gata 9, Malmö, SE-20502 Sweden; 2 Department of Radiation Protection, Swedish Radiation Safety Authority, Stockholm, Sweden

**Keywords:** Utilisation, Radiation dose, Radiation protection, Trends, Population dose, National survey

## Abstract

**Background:**

Diagnostic imaging is a dynamic medical field. In nuclear medicine, advancements introduce new procedures utilising innovative radiopharmaceuticals. These developments may influence supply requirements and exposure levels for the patient population. Surveying the frequency of procedures, types of pharmaceuticals, and administered activities provides valuable insights into utilisation trends and radionuclide demand. This knowledge also guides the prioritisation of radiation protection efforts at national and local levels. In Europe, radiation dose assessments for medical exposures are mandatory according to the directive´s requirements.

**Methods:**

This study evaluated the utilisation of diagnostic nuclear medicine procedures in Sweden over 15 years (2008–2023), focusing on procedure frequency, effective dose, and collective effective dose. Comprehensive data from all Swedish clinics performing nuclear medicine were analysed, incorporating information on radiopharmaceuticals and administered activities. The method suggested by the UNSCEAR, which includes so-called essential procedures, was used for comparison. The study also investigated some frequent procedures in more detail.

**Results:**

The study identifies noteworthy trends, including a threefold increase in the number of clinics offering Positron Emission Tomography (PET) procedures and a significant rise in PET usage. PET procedures constituted over 50% of the collective effective dose for adults in 2023. Despite this, Gamma Camera (GC) procedures still dominate in frequency but exhibit a steady decline. Procedures using ^99m^Tc and ^18^F accounted for 93% of procedures in 2023. The collective effective dose rose 22% over the study period, with PET procedures driving this increase. PET procedures increasing role became evident by the increased contribution to the total collective dose from 15 to 52%. The UNSCEAR methodology captured 67% of the total frequency and underestimated the collective effective dose by 16%. Administered activity remained stable for the selected procedures and showed low variation between clinics.

**Conclusions:**

PET procedures are increasing in scope and now constitute the largest contribution to radiation dose, and in-house production of PET radiopharmaceuticals is available in around 40% of clinics. The number of radionuclides decreased over the study period, and GC procedures declined. In general, the amount of administered activity remained stable over the period for the procedures studied. Accurately assessing utilisation and exposure trends requires extensive data, and the methodology used affects the result significantly.

## Background

In nuclear medicine, radiopharmaceuticals are essential for anatomical, functional, and molecular imaging, playing a pivotal role in medical diagnostics. Research and developmental outcomes may substantially influence utilisation patterns by introducing novel radiopharmaceuticals, using new radionuclides and substances. The production of radionuclides used for Positron Emission Tomography (PET) has been emerging in the last decades [1] and an increasing number of hospitals have access to in-house production of PET nuclides. At the same time, there have been concerns about shortages of radionuclides after production facilities have been decommissioned [2]. Concomitantly advancements regarding other diagnostic modalities such as computed tomography and magnetic resonance imaging may also influence the utilisation of some nuclear medicine procedures and thus change the use of radiopharmaceuticals with time. Consequently, such factors may alter the utilisation of nuclear medicine procedures and this in turn affects radionuclide needs as well as the patient population exposure over time. To understand the levels and trends of exposure resulting from nuclear medicine interventions, comprehensive studies must be conducted.

Studying utilisation of different radiopharmaceuticals requires reliable data from the hospitals defined by medical purpose (indication), used radiopharmaceutical and administered activity. Radiation dose estimates of nuclear medicine procedures are facilitated by the ICRP’s system, which provides standardised methods for assessing effective doses from radiopharmaceuticals [3]. In radiation protection, applying the model and derived factors for reference individuals is regarded as the gold standard. These models are considered to be free from uncertainties [4], but it should be emphasized that for individual patients the organ dose will vary due to inter-person differences.

Basic quantities in studies and analyses of exposure from ionising radiation are the frequency of the procedures, together with the effective dose per procedure. Furthermore, collective effective dose – the product of the two quantities – was introduced used to represent population dose [5]. The United Nations Scientific Committee on the Effects of Atomic Radiation (UNSCEAR) [6] and European commission [7] have conducted investigations to assess radiation exposure and trends over time. In this international context, strategies have been proposed to assess radiation dose and collective effective dose with more limited information compared to if gross data from all clinics performing nuclear medicine procedures are available [8]. A few studies have since then been published presenting the trends in this field [9–14]. Only a few reflect recent advancements in Europe and include country-based studies on population dose [9, 10].

Detailed information on the frequency of procedures and radiopharmaceuticals gives general knowledge of utilisation trends. This facilitates advice on the prioritisation of radiation protection activities at national or local levels. It could also reveal differences in resources and use habits that could improve clinical usage and identify future needs for the production of radionuclides. In Europe, radiation dose assessments for medical exposures are also stipulated by the European Basic Safety Standards Directive [15].

The main objective of this study was to investigate current levels and potential changes of exposure in nuclear medicine practices in Sweden over 15 years. The focus was on procedure frequency, effective dose and collective effective dose and to analyse more detailed data for the most frequent procedures. A secondary goal was to compare the results obtained using the methods recommended by international organisations.

The study uses comprehensive data on procedure and average administered activity from specific examination types collected from all hospitals in Sweden.

## Methods

### The database creation, assessments and analysis

In this study, data sourced from all hospitals conducting diagnostic nuclear medicine examinations in Sweden were utilised and data from the years 2008, 2013, 2018 and 2023 was included in the study database. The latest available data was from 2023. For the study, a five-year interval back to 2008 was selected, as this is assumed to reflect modern practices, including PET procedures. The data encompass details on radiopharmaceuticals (both radionuclide and substance), the number of administrations, and the average administered activity for each specific radiopharmaceutical. The number of administrations is assumed to be equal to the number of procedures. The data was stratified for adults and children (aged below or equivalent to 15 years). The examinations were also grouped into two general groups including diagnostic procedures using gamma cameras (GC), SPECT or SPECT/CT and other detectors into one group and PET, PET/CT and PET/MR into another. This followed the recommendation by the UNSCEAR [7]. The two groups were named GC procedures and PET procedures. This means that a smaller number of procedures such as sentinel node or thyroid uptake using other detector devices was placed in the GC group. The assessment of a typical effective dose was derived for each clinic and radiopharmaceutical was included in the study database. The effective dose was determined using the reported average administered activity. The assessment of effective dose primarily relies on the ICRP conversion factors between administered activity and effective dose [16–19]. In instances where these factors were not available, the conversion factor provided in the product specifications of the radiopharmaceutical published by the Medical Products Agency [20] or, in some cases, scientific publications [21–30] were utilised. The conversion factors for a child of 5 years were used, as the exact mean age of the children was not known. The total collective effective dose was calculated using the data from each radiopharmaceutical at each clinic. The number of inhabitants for children and adults was derived from the database of Statistics Sweden (SCB) [31].

Based on this derived study database, data were analysed to show current utilisation, radiation doses and possible changes over time. The extensive data collected from each clinic gives the clinic’s relative contribution to the national average. The following data was derived. Data on the number of nuclear medicine clinics, the types of radionuclides used, and the overall frequency of procedures, including specific frequencies for Gamma Camera (GC) and Positron Emission Tomography (PET) procedures. Values for adults and children were also derived. The total collective effective dose was derived for subgroups. Subsequently, frequency and collective effective dose ​​were weighted for the number of inhabitants.

Three procedures for adults and three paediatric procedures were studied in more detail. The three most frequent procedures in the year 2023 were chosen for the adult group. That is, oncology procedures using ^18^F-FDG, cardiovascular procedures using ^99m^Tc-tetrofosmin and bone scintigraphy using ^99m^Tc-MDP/HDP for adults were studied. Renal imaging procedures, ^99m^Tc- DMSA and ^99m^Tc- MAG3, were studied, as these were paediatric frequent GC procedures. Oncology procedures using ^18^F-FDG were also studied to include a PET procedure. The frequency, median administered activity and typical effective dose were derived, together with the ratio between the 1st and 3rd quartile (Q1 and Q3) of the administered activity to represent the distribution. The collective effective dose for each study year and procedure was also assessed.

### Comparison with the method used by UNSCEAR

International and national studies have been based on different methodologies. The basic strategy for the present study was to use data for all procedures in all clinics. The method used in the most recent Global survey of the UNCSEAR [7] suggests collecting data from a more limited set of procedures. The manual of the UNSCEAR Global survey indicated some procedure types that are essential to include. This survey groups diagnostic procedures using gamma cameras, SPECT or SPECT/CT and other detectors into one group and PET, PET/CT and PET/MR into another. Within these groups, the procedures were divided into anatomical regions/medical conditions together with a specific radionuclide for each region [7]. These regions provide the basis for showing overall trends. The groups were nervous system, skeletal, cardiovascular, pulmonary, endocrine, gastrointestinal, genitourinary, oncology, infection/inflammation and lymphatics. The typical effective dose for adults was used in the calculations because the frequency for adults was dominant but with one exception: renal procedures. In that case, a weighted typical effective dose including children was derived for renal procedures. The frequency of each group, the relative frequency of the group and the collective effective dose were calculated. The values were not weighted with respect to the frequency of each clinic. A weighted collective effective dose was also calculated as the sum of the collected effective dose for each clinic. The stratification used in the UNSCEAR survey gives an overview of nuclear medicine in general.

### Limitations, uncertainties and strength

The study exhibits limitations and uncertainties comprising several sources. The total dose from hybrid examinations was not derived, i.e. the exposure from computed tomography was not included in the radiation dose estimation. The radiation dose from optimised protocols for attenuation corrections comprises only a minor part of the total exposure but different CT usage may lead to a substantial underestimation of the total radiation dose from the procedure if not taken into account. The lack of information on the mean age of the paediatric patients and the assumption of an average of 5 years introduces uncertainties in the estimations of effective doses for children. This will introduce uncertainties in the calculated effective dose for children. The effective dose may be overestimated if the average age is older than five years and underestimated if it is younger than five years. However, since the main aim of the study is to analyse trends on an aggregated level, this will not significantly affect the overall results. The uncertainty regarding the nomenclature of groups of procedures is a source of uncertainty. The uncertainty in the reported administered activity leads to uncertainty in the effective dose. The overall accuracy of the administered activity value depends on several factors, such as the measurement of the syringe in the activity meter and the residual activity in the syringe. The level of error introduced may vary significantly between different pharmaceuticals, syringe types, and administration methods [32, 33]. While this is difficult to correct for in a national study, it is important to consider in studies aiming to reflect the administered activity for individual patients. The combined uncertainty from these two sources is minor at an aggregated level but becomes more significant when examining detailed data for specific groups. The coverage of the survey was considered a strength of the study and no extrapolation for missing data was needed. Therefore, a rough estimate of the uncertainty of European population dose for NM procedures was estimated using the method set out by the European study [7] to be 5.6% assuming a relative contribution of the factors frequency 5%, mean activity 10% and conversion factor 20%.

## Results

### Frequency, effective dose and collective effective dose - general trends

The number of reporting clinics remained consistent throughout the period (Table 1). However, one regional hospital and one cancer-focused clinic began offering nuclear medicine procedures, bringing the total to 34 clinics in 2023. The number of clinics performing PET procedures tripled over the period. By the end of the period, 13 clinics were conducting PET procedures, and six clinics were producing PET radiopharmaceuticals in-house.

**Table 1:** Number of clinics, frequency of procedures, nuclides and procedures per radionuclide for the study years 2008, 2013, 2018 and 2023 respectively

Year	2008	2013	2018	2023
Number of clinics	32	32	32	34
Clinics performing PET	10	11	11	13
	**Frequency**			
Total	102 347	102 528	110 616	115 879
GC	95 937	88 584	84 680	76 879
PET	6 410	13 944	25 936	39 000
Adults	96 376	96 720	106 245	111 992
GC	90 018	82 868	80 441	73 298
PET	6 358	13 852	25 804	38 694
Children	5 971	5 808	4 371	3 887
GC	5 919	5 716	4 239	3 581
PET	52	92	132	306
	**Number of inhabitants**			
Total	9 256 347	9 644 864	10 230 185	10 551 707
Adults	7 591 566	7 899 577	8 296 079	8 622 713
Children	1 664 781	1 745 287	1 934 106	1 928 994
Number of radionuclides	15	18	13	9
	**Frequency per radionuclide**			
^99m^Tc	83 940	78 071	76 733	72 875
^131^I	3 153	2 560	2 332	2 176
^123^I	1 013	1 351	1 616	1 366
^18^F	6 073	12 377	23 042	34 800
^68^Ga	0	443	1 736	2 759
^11^C	304	964	908	777
^15^O	28	139	180	664
GC other^1^	7 831	6 602	3 999	462
PET other^2^	5	21	70	0

1: (^14^C, ^51^Cr, ^55^Fe, ^59^Fe, ^3^H, ^125^I, ^111^In, ^75^Se, ^201^Tl, ^133^Xe)

2: (^13^N, ^64^Cu)

The number of radionuclide types has decreased during the period (Table 1). In 2023, 9 radionuclides were used, compared to 15 in 2008. Examples of radionuclides that are not used in 2023 are ^14^C, ^51^Cr, ^201^Tl and ^133^Xe. In the period a number of new radionuclides were introduced of which one, ^68^Ga, is still in use in 2023. In 2023, 93% of the procedures were performed with either ^99m^Tc or ^18^F.

**Fig. 1:**
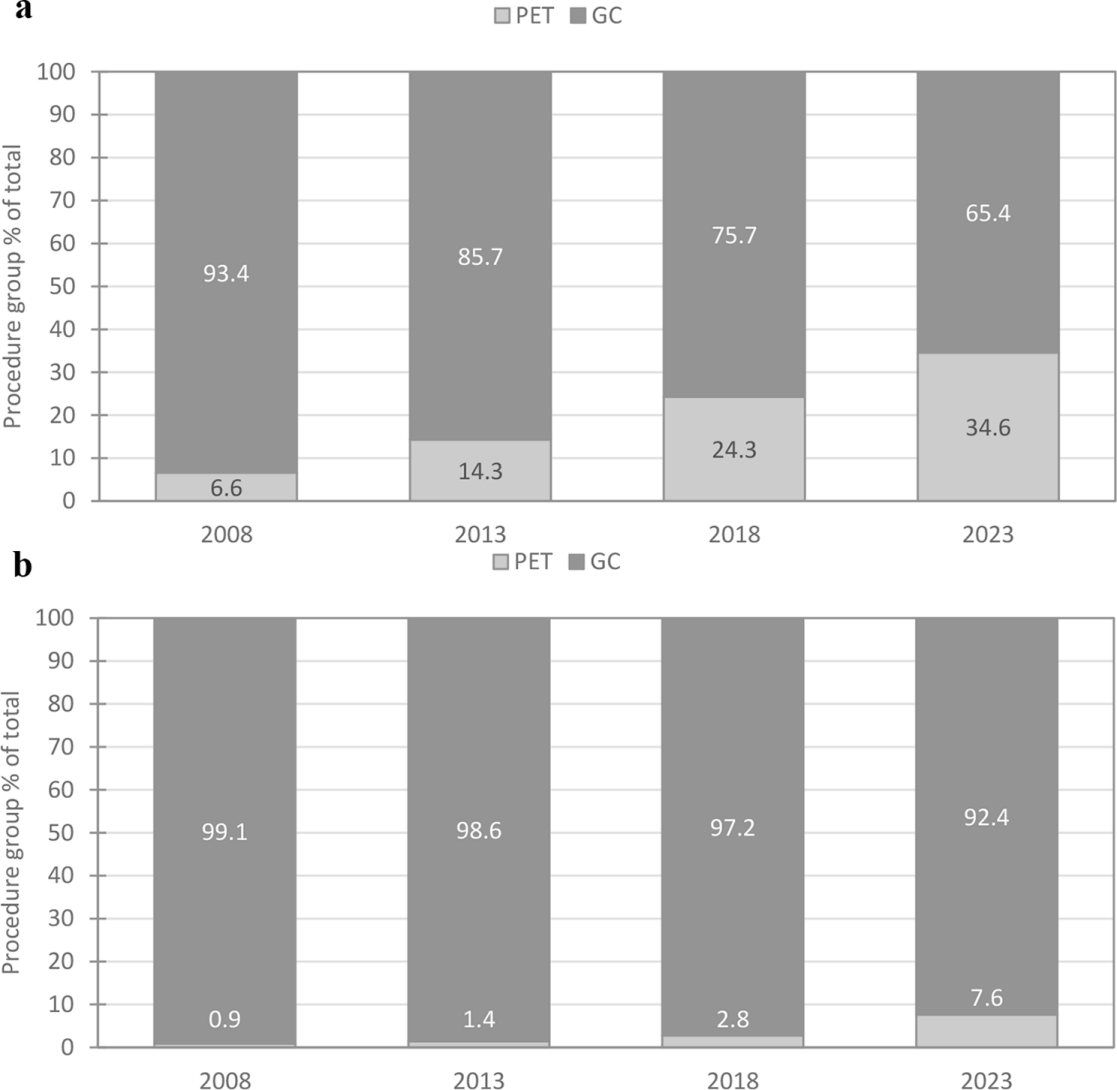
**a**: Percentage of GC and PET procedures, adults. **b**: Percentage of GC and PET procedures, children

The number of GC procedures steadily decreased throughout the period (Table 1), resulting in a slight overall increase in frequency due to the rise in PET procedures. Paediatric procedures accounted for about 10% of the total. A similar trend of declining GC and increasing PET procedures was observed for children. As a result, the percentage of PET procedures relative to the total increased steadily over the period for adults (Fig. 1a). In 2023, PET procedures made up 34.6% of the total, compared to 6.6% at the start of the study. A similar increase was seen in children (Fig. 1b), with PET procedures rising from 0.9 to 7.6% in 2023.

**Fig. 2:**
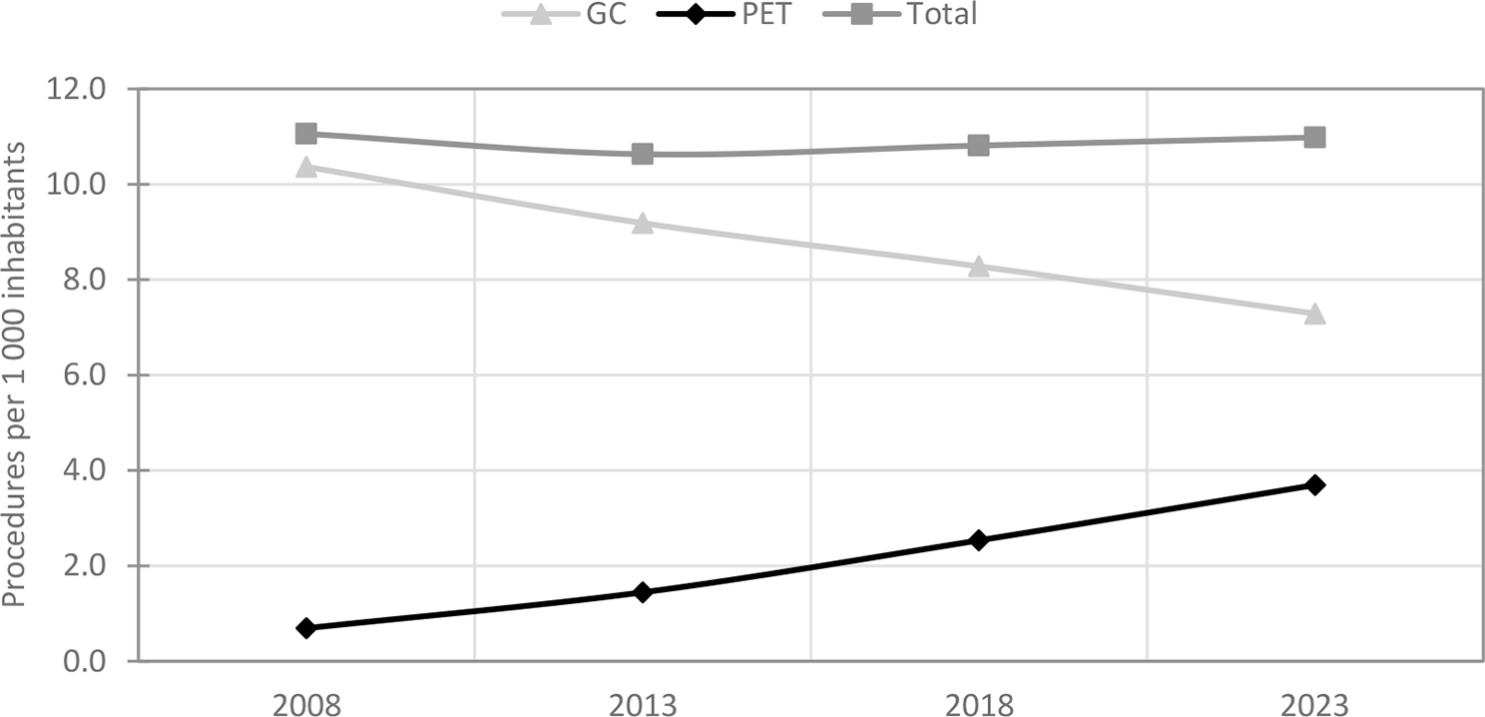
Procedures per 1000 population trends: total, PET, and GC procedures respectively

Overall trends in procedures per 1000 population are shown in Fig. 2, with separate values for GC and PET procedures. In 2023, the total frequency was 11 per 1000 population. GC procedures decreased by 30%, while PET procedures increased by 434%, accounting for population size.

**Fig. 3:**
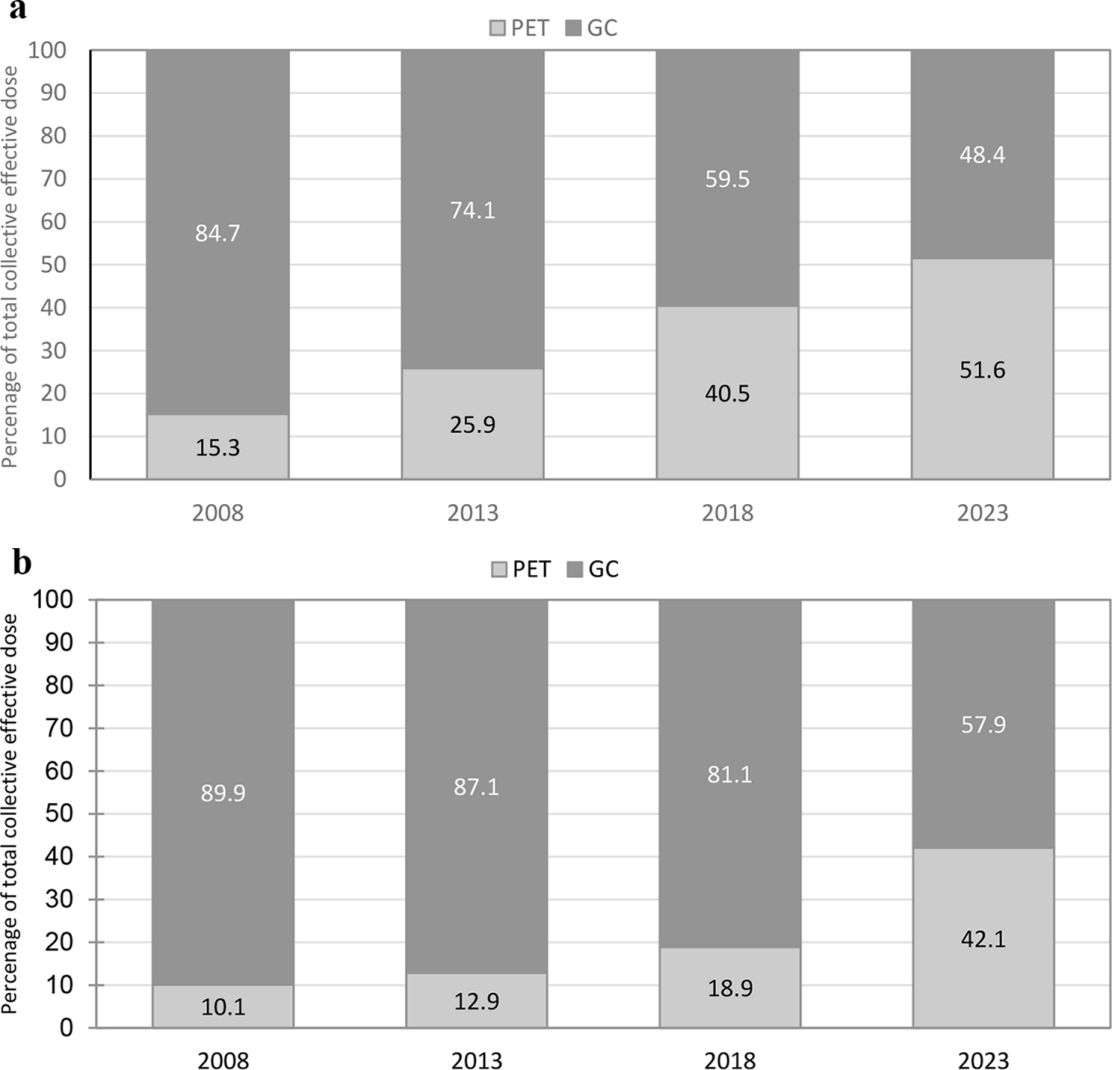
**a** Percentage values of collective effective dose (person Sv) for PET and GC procedures, adults. **b** Percentage values of collective effective dose (person Sv) for PET and GC procedures, children

The estimated collective effective dose is presented in Table 2. The collective effective dose increased from 289 to 351 person Sv in the period. The collective effective dose aligns with the trend in procedure frequency; as GC procedures decrease in prevalence; PET procedures contribute a relatively greater proportion. The contribution to the collective effective dose from PET procedures in 2023 was 51.5% which is an increase from 15.1% in 2008. The percentage collected effective dose from GC and PET procedures is shown in Fig. 3a and b for adults and children respectively. The percentage of collective effective dose from PET procedures for adults is 51.6% in 2023 being 15.3% in 2008. The number of PET procedures for children was lower than for adults, with the percentage of the total collective effective dose accounting for 42.6% in 2023.

**Fig. 4:**
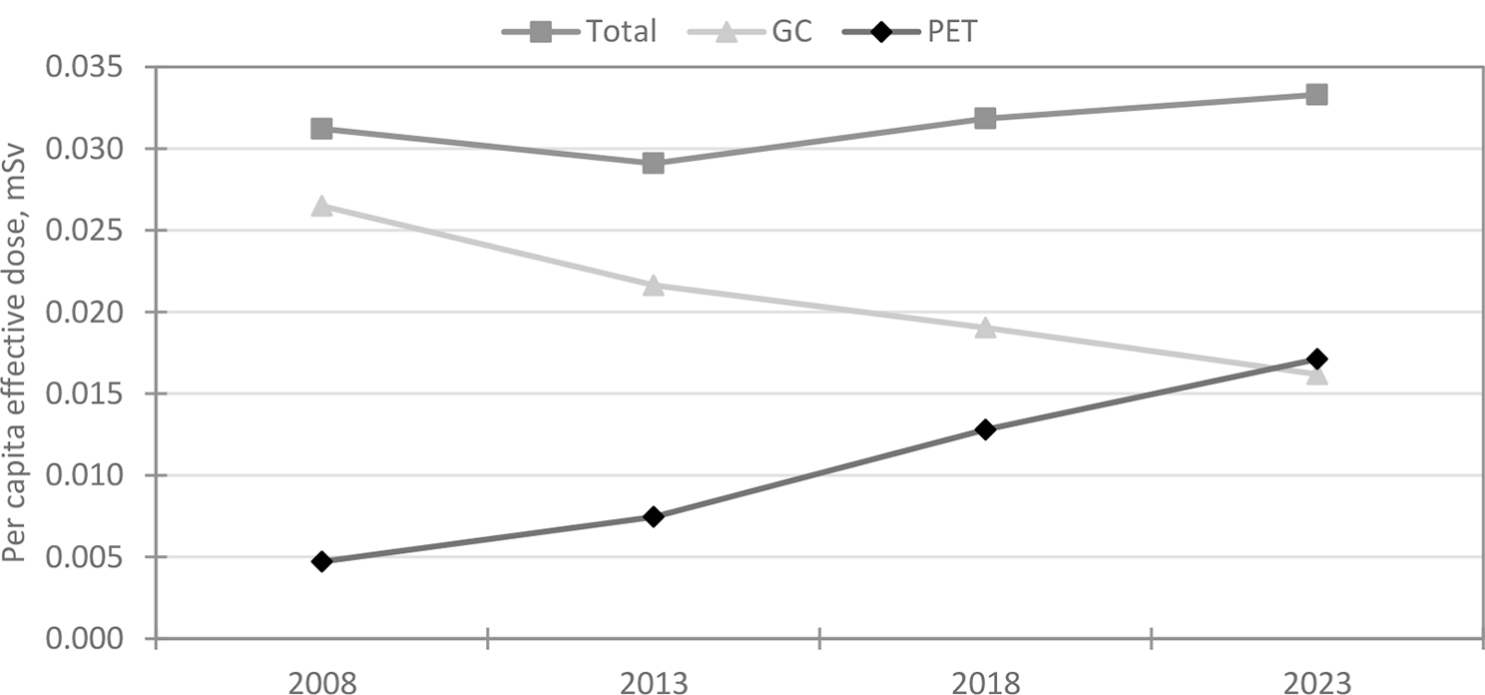
Per capita effective dose (mSv) trends

The per capita effective dose is shown in Fig. 4. The per capita effective dose is 0.033 mSv in 2023 and the contribution from PET procedures is 0.017 mSv. The respective values for 2008 were 0.031 mSv and 0.005 mSv.

**Table 2:** Total collective effective dose (S), person Sv, and S per procedure group

Year	2008	2013	2018	2023
	**Collective effective dose**,** person Sv**			
Total	289	281	332	351
PET	44	72	131	181
GC	245	209	201	170
Adults total	282	274	328	346
Adult PET	43	71	130	179
Adult GC	239	203	198	167
Children total	7.1	6.2	4.4	5.7
Children PET	0.7	0.9	0.9	2.4
Children GC	6.4	5.3	3.5	3.3
	**Collective dose person Sv per 1000 population**			
Total	31.2	29.1	32.5	33.3
PET	4.7	7.5	12.8	17.1
GC	26.5	21.6	19.7	16.2
	**Per caput effective dose**,** mSv**			
Total	0.031	0.029	0.032	0.033
PET	0.005	0.007	0.013	0.017
GC	0.026	0.022	0.019	0.016

### Specific data for some common procedures

Detailed data (frequency, median administered activity, effective dose and collective effective dose) of the three most frequent adult procedures (defined by the purpose of the procedure and radiopharmaceutical) and frequent paediatric procedures are given in Table 3.

**Table 3:** The three most frequent procedures for adult and the two frequent procedure for children in the year 2013 and key exposure parameters during the years

Year	2008	2013	2018	2023
	**Adult**			
	**Oncological procedure** ^**18**^ **F-FDG**			
Frequency	5 790	11 196	19 873	28 074
Median administered activity, MBq	319	298	292	239
Ratio Q1/Q3	1.31	1.10	1.28	1.18
Median effective dose, mSv	6.1	5.7	5.5	4.5
Collective effective dose, personSv (% of total)	41 (15%)	62 (23%)	104 (32%)	137 (40%)
	**Cardiovascular**, ^**99m**^**Tc-tetrofosmin**			
Frequency	16 143	17 613	23 055	25 499
Median administered activity, MBq	538	556	513	476
Ratio Q1/Q3	1.36	1.25	1.29	1.31
Median effective dose, mSv	4.0	4.2	3.8	3.6
Collective effective dose, personSv (% of total)	72 (26%)	74 (27%)	85 (26%)	85 (25%)
	**Bone scintigraphy**, ^**99m**^**Tc-MDP/HDP**			
Frequency	21 875	17 436	15 522	13 061
Median administered activity, MBq	499	538	548	554
Ratio Q1/Q3	1.36	1.13	1.10	1.12
Median effective dose, mSv	2.4	2.6	2.7	2.7
Collective effective dose, personSv (% of total)	54 (19%)	45 (16%)	41 (13%)	35 (10%)
	**Paediatric procedures**			
	**Oncological procedure** ^**18**^ **F-FDG**			
Frequency	51	77	109	253
Median administered activity, MBq	265	172	127	129
Ratio Q1/Q3	1.28	1.78	1.29	1.20
Median effective dose, mSv*	14.9	9.6	7.1	7.3
Collective effective dose, personSv (% of total)	0.72 (10%)	0.75 (12%)	0.72 (16%)	2.1 (38%)
	**Renal imaging**, ^**99m**^**Tc-DMSA**			
Frequency	3 029	3 411	2 407	2 187
Median administered activity. MBq	30	29	31	28
Ratio Q1/Q3	1.64	1.76	1.46	1.36
Median effective dose. mSv*	0.6	0.6	0.7	0.6
Collective effective dose, personSv (% of total)	2.0 (28%)	2.5 (40%)	1.7 (38%)	1.3 (22%)
	**Renal imaging**, ^**99m**^**Tc-MAG3**			
Frequency	1 045	961	967	879
Median administered activity. MBq	32	31	31	33
Ratio Q1/Q3	2.38	1.60	1.30	1.29
Median effective dose, mSv*	0.38	0.37	0.37	0.40
Collective effective dose, personSv (% of total)	0.47 (6.7%)	0.43 (6.9%)	0.37 (8.3%)	0.35 (6.1%)

* 5 year old

The three procedures constitute 60% of the number of adult procedures. The oncology procedures using ^18^F-FDG have increased in number as expected, from 0.8 per 1000 population to 3.3 per 1000 population. The median effective dose decreased somewhat during the period and shows a narrow interquartile range. That is, the clinics use similar amounts of administered activity. The percentage of the total collective effective dose was 40%, constituting the majority of the collective effective dose from PET procedures. The cardiovascular procedure using ^99m^Tc-tetraphosmin has also increased from 2.1 per 1000 population to 3.0 per 1000 population. The median effective dose has slightly decreased, with similar amounts of administered activity used across clinics for this procedure. The percentage of the total collective effective dose was 25% and has been at the same level during the period. Bone scintigraphy using ^99m^Tc-HPD/MPD has decreased from 2.9 per 1000 population to 1.5 per 1000 population. The median effective dose has slightly increased. The percentage of collective effective dose from bone scintigraphy has decreased from 19 to 10% of the total collective effective dose during the period. These three procedures are the most frequent and contribute the most to the total collective dose. Other procedures contribute less than 2% to the total collective effective dose.

For paediatric procedures, it is more challenging to identify those that significantly contribute to the collective effective dose. The three procedures chosen constitute 85% of the total number. The oncology procedures using ^18^F-FDG are the most frequent PET procedure for children. This procedure has increased from 0.03 per 1000 population to 0.13 per 1000 population. The median administered activity was at its highest in 2008 and decreased distinctly to 2013, but for 2018 and 2023, the level is similar. The resulting decrease in effective dose led to the same level of collective effective dose due to the increased number. The renal procedure using ^99m^Tc-DMSA is the most frequent paediatric procedure. The frequency ranges from 1.8 per 1000 population and 1.1 per 1000 population in the period, i.e. a substantial decrease. Throughout the period, the administered activity remained consistent, resulting in the effective dose remaining unchanged. The decreased frequency has lowered the collective effective dose. The contribution to the collective effective dose to children from renal using ^99m^Tc-DMSA is 22% in the year 2023. The renal examination using ^99m^Tc-MAG3 contributes much less to the collective effective dose due to the lower typical effective dose and shows similar trends regarding decreased frequency as renal imaging using ^99m^Tc-DMSA.

### Comparison with UNSCEAR methodology

The results obtained using the UNSCEAR-specified method are presented in Table 4. The procedures deemed essential by UNSCEAR account for 67% of the total number of procedures. This leads to a 16% underestimation of the assessed collective effective dose. This discrepancy could be reduced by incorporating pulmonary and oncological PET procedures, such as those performed with ^68^Ga, into the evaluation. The difference between the weighted collective dose (the result of the present comprehensive collection) and the unweighted at the aggregated level is 15%. However, for a specific group the difference could be both positive and negative. The largest difference amounted to nearly 80%. The largest difference was observed in GC cardiovascular and PET oncology procedures. The contribution of the group GC oncology procedures and the group infection/inflammation is low. The UNSCEAR compilation also identifies some groups that are frequent but contribute to the collective effective dose to a lesser extent, e.g., lymphatics.

**Table 4:** Frequency, relative frequency, typical effective dose and collective effective dose S (unweighted and weighted) for the study year 2023 using the UNSCEAR method. The essential groups indicated by UNSCEAR is defined

Group	Radionuclide	Number	Typical effective dose mSv	Percentage frequency %	S unweighted person mSv	S weighted person mSv
GC nuclides						
Nervous system	^99m^Tc	159	5.8	0.2	922	893
Nervous system	^123^I	1 141	4.5	1.5	5123	5 129
Skeletal (essential)	^99m^Tc	13 133	2.6	17.1	34 188	35 539
Cardiovascular (essential)	^99m^Tc	27 950	3.5	36.4	99 125	95 679
Cardiovascular (essential)	^201^Tl			0.0		
Pulmonary	^99m^Tc	7 986	1.2	10.4	9 901	8 218
Endocrine (essential)	^99m^Tc	6 068	3.8	7.9	23 147	14 826
Endocrine (essential)	^123^I	128	4.9	0.2	632	775
Gastrointestinal	^99m^Tc	632	2.2	0.8	1 390	545
Genitourinary	^99m^Tc	7 242	0.5	9.4	3 745	4 203
Oncology	all	366	3.6	0.5	1 302	568
Infection. inflammation	^99m^Tc	104	4.7	0.1	493	528
Lymphatics	^99m^Tc	9 171	0.5	11.9	4 329	2 840
Thyroid uptake	^131^I	2 169	0.01	2.8	24	43
Other	^99m^Tc	146	4.5	0.2	662	381
Other	^75^Se	453	0.3	0.6	122	155
Other	^111^In, ^123^I, ^131^I	31	4.1	0.0	126	132
PET nuclides						
Oncology (essential)	^18^F	30 262	6.1	77.6	184 613	146 898
Oncology	^68^Ga	2 759	4.8	7.1	13 167	8 539
Cardiology	^18^F					
Cardiology	^15^O	664	0.4	1.7	292	292
Skeletal	^18^F	83	2.2	0.2	181	363
Nervous system	^18^F	4 039	4.6	10.4	18 623	22 046
Infection. inflammation	^18^F					
Nervous system	^11^C	693	2.6	1.8	1 769	966
Oncology	^11^C	82	2.4	0.2	194	187
Other	^11^C	2	2.2	0.0	4	4
Other	^18^F	416	4.3	1.1	1 785	1 809
Total		115 879			351 558	405 858
GC essential		47 279			146 819	157 091
PET essential		30 262			146 898	184 613
Total essential		77 541			293 717	341 705

## Discussion

This study investigates the utilisation of diagnostic nuclear medicine procedures in Sweden, based on comprehensive data from all clinics nationwide. The frequency and collective effective dose were analysed to provide an overview of the practices over the past 15 years. The overall frequency and collective effective dose remained relatively stable between 2008 and 2023, with the number of clinics also remaining consistent. However, the study indicates a slight increase in the collective effective dose, primarily due to the rise in PET procedures. There has been a notable shift in the utilisation of specific procedures, with an increased percentage of PET procedures contributing more significantly to the collective effective dose. This trend may also lead a rise in the collective effective dose per capita. The growing number of clinics performing these procedures, with 13 out of 34 clinics now offering PET procedures has likely facilitated the increase in procedures, suggesting a potential further elevation in the coming years.

The number of radionuclides in use has decreased over time. A temporary increase observed in the middle of the study period was attributed to the introduction of certain PET nuclides, which were used for a few years before being phased out, alongside the continued use and subsequent discontinuation of some radionuclides. Two PET nuclides introduced during this period − ^68^Ga and ^15^O - appear to remain important. In 2023, about 93% of diagnostic nuclear medicine procedures utilised ^99m^Tc or ^18^F. Approximately 45 different radiopharmaceuticals are still in use, presenting ongoing challenges for accurate radiation dose assessments.

Comparisons with other countries are valuable; however, recent studies are scarce. Current Swedish data can be compared with Swiss and Finnish studies [9, 10], both of which include data from 2018. Unfortunately, the data presented in the UNSCEAR study is older, reducing the relevance of such comparisons. The findings of the Swiss study, reporting a rate of 13.3 per 1 000 population, are higher than the Swedish value of 10.8 per 1 000 population. The effective dose per capita was 0.107 mSv in Switzerland, substantially higher than the 0.033 mSv determined in the present study. This discrepancy may be attributed to the average effective dose from nuclear medicine procedures in Switzerland, which was reported to be 8.04 mSv, a notably higher figure compared to the observed mean value in this study 2.7 mSv. In Finland, the collective effective dose for 2018 was estimated at 215 person Sv, resulting in a per capita effective dose of 0.04 mSv. This value is slightly higher than the corresponding Swedish figure, 0.03 mSv. Both the Swiss and Finnish studies included the CT component of hybrid procedures. The Finnish values indicate that the differences observed between the Swiss and Swedish studies are not solely attributable to the absence of the CT component in the Swedish study. Additionally, the proportion of PET examinations in Finland was 28%, compared to 24% in Sweden in 2018.

The study conducted separate analyses for adult and paediatric groups and used the size of the population for the two groups separately in the assessment. The contribution of paediatric procedures to the total collective effective dose is low due to their infrequent occurrence. If data collection for paediatric cases proves challenging, it may be reasonable to use the total frequency and effective dose values derived from adult cases instead. The uncertainty in effective dose estimation was also higher for the paediatric group, primarily because the average age for different procedures was not collected. The UNSCEAR Global survey indicates that patient age distribution also should be collected. Since the effective dose is derived rather than radiation risk, knowing the contribution of different age subgroups within the adult population seems less relevant. However, the age distribution for frequency is useful if radiation risk values are derived, but the collective effective dose may be derived independently of age. However, the age distribution in a country can significantly affect the intervention rate due to the morbidity correlation with age. This affects the comparisons between countries.

When examining radiation doses in detail, it is crucial to focus on a specific medical indication using a particular radiopharmaceutical. An analysis of the three most common adult procedures revealed several key observations. The amount of administered activity remained relatively consistent over the observed time. Furthermore, the range of administered activity per procedure is quite narrow, as indicated by the ratio between the third and first quartiles. This suggests that collecting administered activity data from all clinics may not be necessary.

Interpreting data on paediatric procedures is challenging due to statistical limitations, particularly the low and variable number of procedures performed annually. Nevertheless, a relative decline in the frequency of renal imaging has been observed, leading to a greater proportional contribution of other GC procedures, such as thyroid scintigraphy, to the total number of paediatric procedures.

Comparative analyses between countries are to some extent feasible given the availability of data related to Diagnostic Reference Levels (DRLs). In Sweden national diagnostic reference levels were established in 2002 and since then revised twice. Administered activity for the three studied procedures is well in line with the current reference values. A recent French study [34] has reported median activity levels across a broad spectrum of hospitals, encompassing both adult and paediatric procedures. For bone scintigraphy using ^99m^Tc-HDP, the median administered activity was 662 MBq, with a quartile ratio of 1.13. In cardiology, utilising ^99m^Tc-tetrophosmin, median activity levels were recorded at 550 MBq for rest and 553 MBq for stress procedures, with a quartile ratio of 1.45. For ^18^F-FDG procedures, the median activity was 191 MBq, with a quartile ratio of 1.35. In the present study, the observed activity levels are generally lower, except in the case of ^18^F-FDG procedures. The French study also stratified paediatric patients into five weight categories, ranging from 5 to 55 kg. Median activity values for renal cortical procedures varied from 23.9 to 67.7 MBq across these weight groups, with quartile ratios ranging from 1.20 to 1.57. The Swedish data appear at comparable or slightly lower levels, with a similar quartile ratio of 1.27. Reported activity levels for ^18^F-FDG procedures in the French cohort varied between 68.7 and 140 MBq, with quartile ratios between 1.07 and 1.26. Notably, in the present study, the median activity for ^18^F-FDG is higher, at 253 MBq, with a comparable quartile ratio of 1.15.

A recent study from Austria [35] on DRLs has reported values consistent with those observed in France. For bone scintigraphy with ^99m^Tc-HDP the median activity was 663 MBq, while for ^18^F-FDG procedures, the median was 197 MBq. The findings for myocardial procedures were also aligned with the French data. A recent study from Japan reports administered activity for a wide range of different radionuclides in examinations of adults, as well as the three procedures included in this study [36]. The median value for ^18^F-FDG was 220 MBq comparable to that reported in all the previously mentioned countries. The value for ^99m^Tc-tetrophosmin, median activity levels were recorded at 740 MBq about a 30% difference from the values reported from France and Sweden. The median administrative activity of ^99m^Tc-MDP used for bone scintigraphy was also higher in this study compared to the French and Swedish studies.

The UNSCEAR approach was valuable in clarifying the contributions of different groups of procedures. The results indicate that including only essential procedures would significantly underestimate both the overall frequency and the total effective dose. To utilise such data, extrapolation is required, introducing considerable uncertainties. A European study suggested the “Top 7” method as a potential solution when no comprehensive data was available. However, this method was not used in the present study as it became evident that the proposed procedures were no longer relevant. Identifying the seven most relevant procedures would require extensive data, as the selection depends on both the frequency of the procedures and the typical effective dose associated with each. This necessitates comprehensive data collection, raising questions about the practicality and benefits of the proposed method.

To describe the utilisation of nuclear medicine and associated radiation doses, it is essential to analyse multiple data sets and values. Relying solely on metrics like the collective effective dose may provide an inadequate overview. Total frequency can help assess equipment needs, radionuclide usage and production demands. The distribution of administered activity could reflect the optimisation of radiation protection measures. Additionally, comparisons at the European level could provide valuable insights but would require standardised methods. While some comparisons are already feasible, harmonising methodologies would enhance their reliability and depth.

## Conclusions

The key metrics of current practice include 115 879 procedures, representing a 13% increase from 2008 to 2023. The number of procedures per 1 000 population fluctuated during the study period, influenced by population growth. The collective effective dose reached 351 person Sv, marking a 22% increase, corresponding to a per capita effective dose of 0.033 mSv, indicating a 7% rise during the study period.

The practice has evolved, with current trends showing that over one-third of procedures now involve PET, a modality that has experienced continuous growth throughout the study period. Notably, although only 13 out of 34 clinics currently perform PET scans, these procedures contribute over 50% of the collective effective dose.

Throughout the study period, both increases and decreases in procedure frequency were observed. It is not straightforward to determine how these fluctuations affect the overall population dose. Selecting only a few procedures to identify an overall trend is challenging. Data from previous studies may also be misleading when a new study is planned. The study indicates that the administered activity for the procedures remains consistent over time, with low variation also between clinics. Consequently, the number of procedures becomes the key factor in calculating the population dose.

When assessing population exposure from diagnostic nuclear medicine procedures, focusing solely on the most frequent procedures can be problematic, potentially leading to a significant underestimation of exposures. Additionally, utilization rates and the use of different radiopharmaceuticals for the same procedure likely vary across countries. Therefore, comprehensive frequency data are essential for accurate exposure assessment in nuclear medicine. In cases where collecting data on administered activity is more challenging, information from a smaller number of clinics may suffice, as long as it reflects the radiopharmaceuticals in use.

## Data Availability

The datasets used and/or analysed during the current study are available from the corresponding author on reasonable request.

## Abbreviations

GC: Gamma Camera
PET: Positron Emission Tomography
MR: Magnetic Resonance
S: Collective Effective Dose
Sv: Sievert
UNSCEAR: United Nations Scientific Committee on the Effects of Atomic Radiation
CRP: International Commission on Radiological Protection
DRL: Diagnostic Reference Level
Q1: First Quartile
Q3: Third Quartile
